# Successfully treatment of application awake extracorporeal membrane oxygenation in critical COVID-19 patient: a case report

**DOI:** 10.1186/s13019-020-01376-9

**Published:** 2020-12-17

**Authors:** Junyi Tang, Wencan Li, Fanli Jiang, Tao Wang

**Affiliations:** Department of Thoracic and Cardiovascular Surgery, The Affiliated Zhuzhou Hospital Xiangya Medical College CSU, 116 Changjiangnan Road, Zhuzhou, 412007 Hunan China

**Keywords:** Awake extracorporeal membrane oxygenation, COVID-19, Case report

## Abstract

**Background:**

A newly infectious diseases named coronavirus disease 2019 (COVID-19) emerged in China and now has spread in many countries, and constituted a public health emergency of international concern. Extracorporeal membrane oxygenation (ECMO) is used as salvage therapies in critical COVID-19 patients with respiratory/cardiac failure.

**Case presentation:**

A 49-year-old female patient was diagnosed with COVID-19 and progressed to critical cases, she was successfully treated with the application of awake extracorporeal membrane oxygenation. This case is the first reported successfully treatment of application awake ECMO in critical COVID-19 patient in China.

**Conclusions:**

Here we present the first reported successfully treatment of application awake ECMO in critical COVID-19 patient, however, whether awake ECMO can be widely used in the treatment of critical COVID-19 patients need more practice.

## Background

A newly infectious diseases COVID-19 emerged in China and now had spread in many countries, and constituted a public health emergency of international concern. ECMO was used as salvage therapies in critical COVID-19 patients with respiratory/cardiac failure. However, due to the high cost, complex technology and uneven resource distribution, cases of ECMO used in COVID-19 patients were rare. Here we presented the first case of successfully treated critical COVID-19 patient with awake ECMO in China, this report illustrated why, when and how we applying awake ECMO in critical COVID-19 patient.

## Case presentation

**Fig. 1:**
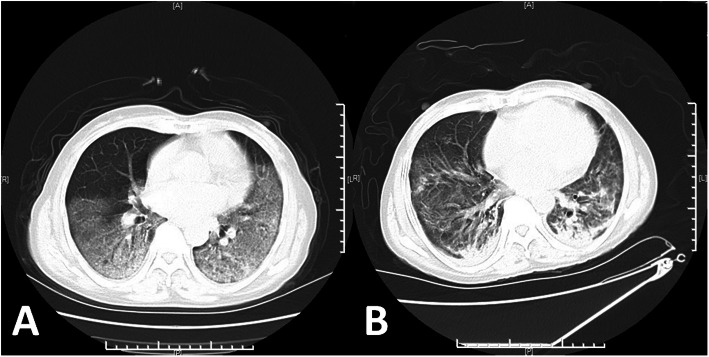
**a** Chest CT on the 8th day of admission. **b** Chest CT on the 39th day of admission

**Fig. 2:**
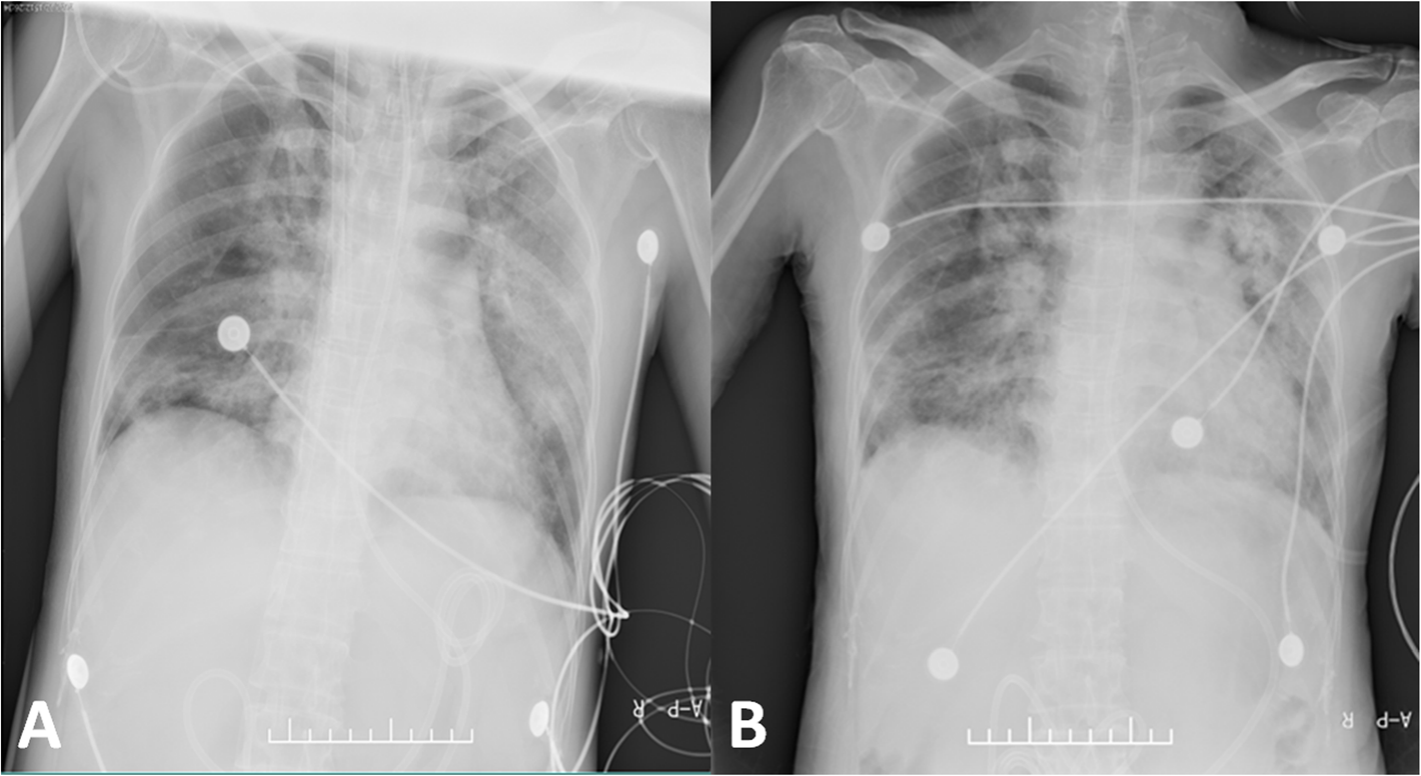
**a** Chest radiograph on the 19th day of admission. **b** Chest radiograph on the 35th day of admission

On January 28, 2020, a 49-year-old female patient was transferred to our hospital for further treatment after being diagnosed with COVID-19. At admission, She presented with fever, cough, sputum, and mild dyspnea. Her body temperature was 38 °C, blood oxygen saturation (SPO2) was 93% under ambient air, oxygen inhalation was immediately given by nasal catheter. After admission, levofloxacin and human immunoglobulin were injected into the veins, recombinant with human interferon a2b atomized inhalation. On the 5th day of admission, the disease deteriorated and her mental state was poor, body temperature was 38.8 °C, blood gas analysis: PO2 49 mmHg, PCO2 38 mmHg. She suffered from type I respiratory failure, which conformed to the diagnostic criteria of severe COVID-19 according to the COVID-19 health guidelines of China national health commission. On the 8th day, oxygen inhalation was given by oxygen storage mask (8 L/min), SPO2 was 93%, blood gas analysis: PO2 49 mmHg, PCO2 38 mmHg, Chest CT indicated multifocal ground glass opacities in both lungs with consolidation in partial lungs, which involving more than 75% of the lungs (Fig. 1a). On the 12th day of admission, the patient was anxious, agitated, Alprazolam was administered for sedation. Under high-flow oxygen inhalation (FiO_2_ 90%, 50 L/min), SPO2 was 83 to 90%, blood gas analysis: PO2 55 mmHg, PCO2 44 mmHg, the oxygenation index<70 mmHg, which indicating poor oxygenation status, and non-invasive positive airway pressure ventilation was immediately performed. On the 14th day of admission, SPO2 was 90%, blood gas analysis: PO2 48 mmHg, pCO2 37 mmHg, under the condition of non-invasive positive airway pressure ventilation, FiO_2_ 70%, which revealed poor and difficult to ameliorate hypoxemia, mechanical ventilation became imperative. The patient progressed to critical cases and was transferred to intensive care unit (ICU), mechanical ventilation was performed by orotracheal intubation, ventilator conditions: Volume Control ventilation, VT 240 ml, VF 15 times/min, FiO2 100%, PEEP 10 cm H2O, prone position ventilation was performed at the same time. After intubation, maintaining the use of propofol and midazolam for sedation, SPO2 rose to 95% and hypoxemia improved. On the 16th day of admission, the patient’s SPO2 was difficult to maintain with poor oxygenation index and high airway platform pressure, salvage VV-ECMO therapy was performed. Under the guidance of B-ultrasound, the right femoral vein was inserted into the inflow cannula, the right jugular vein was inserted into the outflow cannula, the venous cannula was 20F, the arterial cannula was 17F, the depth of venous cannula was 43 cm, and the depth of arterial cannula was 14 cm. Initial ECMO parameters: speed 3200 rpm, flow 5 L/min, Sweep gas 3 L/min, FiO_2_ 70%. Coordinated ventilator parameters: Assist-Control ventilation, VT 210 ml, VF 18 times/min, FiO_2_ 40%, PEEP 12 cm H2O. Reviewed blood gas analysis: PO2 84 mmHg, PCO2 46 mmHg, oxygenation index improved significantly after ECMO. During the treatment of ECMO, deep sedation was performed and heparin was continuously pumped to maintain activated partial thromboplastin time (APTT) being 40–60s. On the 19th day of admission, support condition of ECMO for the patient was still high, ECMO could not be removed in a short time, and the lung compliance was poor. Chest radiograph showed increased multiple patchy density shadows in both lungs (Fig. 2a). We decided to coordinating prone position ventilation to improve pulmonary ventilation-to-perfusion ratio. On the 22th day of admission, bronchoscopy showed: a little white sputum could be seen in the main bronchus, and slightly swelling, hyperemia could be seen in the grade 1–4 bronchial mucosa of both lungs. On the 27th day of admission, the patient was tested negative for SARS-CoV-2 nucleic acid by the fluorescence quantitative RT-PCR for two consecutive times. After the withdrawal of sedative drugs, the patient was conscious, had a firm handshake, we stopped the ventilator, ECMO parameters was adjusted: speed 3600 rpm, flow 4 L/min, Sweep gas 3 L/min, FiO_2_ 70%, oxygen was inhaled through the endotracheal tube whit high-flow oxygen therapy (FiO_2_ 45%, 40 L/min). After observated for 30 min, blood gas analysis: PO2 71 mmHg, pCO2 45 mmHg, heart rate was 83 beats per minute, breathing rate was 25 times per minute, and blood pressure was 136/63 mmHg, the endotracheal tube was removed, awake ECMO was performed. Treatment strategies during awake ECMO stage: 1. Strengthen the monitoring and management of bleeding and thrombosis, monitoring the levels of hemoglobin, platelets, APTT and fibrinogen, and set the corresponding target values to be 90 g/L, 100*10^9/L, 40S, 2.0 g/L respectively, supplement the substrate by transfuse some components of blood if failed to meet target values. 2. Pulmonary rehabilitation: prone position or high lateral lying position was adopted for drainage to promote lung recruitment, and a large dose of ambroxol and acetylcysteine were used to dispersing phlegm. 3. During the awake ECMO period, patients had intermittent anxiety and delirium, enhanced psychological counseling, quetiapine and haloperidol were given to fight anxiety and delirium. 4. Combined Piperacillin tazobactam, Datomycin and Voriconazole to fight infection. 5. Strengthen liquid management and nutritional support therapy. On the 35th day of admission, the patient’s oxygen saturation could be maintained at 98%. After re-examination of chest radiograph (Fig. 2b), the patient was evacuated from ECMO. Reexamination chest CT on March 6, 2020 indicated the ground glass opacities absorbed, and leave some fibrotic stripes (Fig. 1b). After further treatments of anti-infection, pulmonary rehabilitation, nutritional support, psychological counseling and physical rehabilitation, the patient recovered and was discharged on March 15, 2020.

## Discussion and conclusions

From admission, the patient’s oxygenation continues to deteriorate following the progression of disease. On the 16th day of admission, in the state of mechanical ventilation support, measures such as lung protective ventilation strategy and prone position ventilation have been taken, the oxygenation index<100 mmHg, with high airway platform pressure, salvage VV-ECMO was imperative. Eleven days after VV-ECMO, lung compliance of the patient became worsen with lung consolidation, the bronchoscopy showed that there were few airway secretion in large airways, moreover, the first autopsy report of COVID-19 from Wuhan indicated that COVID-19 is a highly infectious disease primarily targeting pulmonary alveoli [1], we decided to remove the endotracheal intubation and perform awake ECMO. Compared to mechanical ventilation, spontaneous breathing can optimize the movement of thoracic and diaphragm muscles, promote the recovery of the ability to expectorate, promote the discharge of essential substances in alveoli and small airways, thus led to the increasing of ventilation-to-perfusion ratio [2].

To our knowledge, we presented the first case of successfully treated critical COVID-19 patient with awake ECMO in China. Patients who have severe respiratory failure, have been invasively ventilated for ≤7 days and meet general guidance criteria without extrapulmonary organ failure may be considered for ECMO [3]. However, due to the high cost, complex technology and uneven resource distribution of ECMO, cases of ECMO used in COVID-19 patients were rare. According to literature review, currently, there were no case reports of critical COVID-19 patients treated with awake ECMO. Past epidemics, ECMO had been applied to the treatment of Middle East respiratory syndrome (MERS) and H7N9 [4, 5], including a case report of a failed awake ECMO for H7N9 [5]. The experiences we learned from this case: 1. For critical COVID-19 patients with ARDS, early applying of mechanical ventilation coordination with prone position ventilation was very helpful to promote lung recruitment, early awake ECMO was inappropriate. 2. For critical COVID-19 patients treated with ECMO, if they experienced mechanical ventilation for a long time or ventilation airway pressure was too high, so long as they were consciously awake in the condition of non-sedation and without multi-organ dysfunction, awake ECMO could promote the recovery of the ability to expectorate and promote lung recruitment. We successfully applied awake ECMO in the treatment of critical COVID-19 patient, but it was just one case in our hospital, more clinical evidences were necessary to verify whether awake ECMO could be widely used in the treatment of critical COVID-19 patients or even severe ARDS caused by other epidemics.

## Data Availability

Not applicable.

## Abbreviations

COVID-19: Coronavirus disease 2019
ECMO: Extracorporeal membrane oxygenation
RT-PCR: Reverse transcriptase polymerase chain reaction
SPO2: Blood oxygen saturation
PO2: Partial pressure of oxygen;
PCO2: Partial pressure of carbon dioxide
FiO_2_: Fraction of inspired oxygen
WBC: White blood cells
NE%: Neutrophil percentage
LY%: Lymphocyte percentage
ESR: Erythrocyte sedimentation rate
CRP: C-reactive protein
TSH: Thyroid stimulating hormone
FT3: Free triiodothyrogenic acid
CT: Computed tomography
ICU: Intensive care unit
VT: Tidal volume
VF: Ventilator frequency
PEEP: Positive end expiratory pressure
APTT: Activated partial thromboplastin time
MERS: Middle East respiratory syndrome
